# Cardiovascular impact of chronic venous disease: A systematic review and meta-analysis

**DOI:** 10.1016/j.ijcha.2026.102024

**Published:** 2026-10-01

**Authors:** Leonardo De Luca, Vanessa Blanc-Guillemaud, Marie-Blanche Onselaer, Soumeya Serifou, Hurrem Pelin Yaltirik, Giuseppe Biondi-Zoccai

**Affiliations:** a Division of Cardiology, Fondazione IRCCS Policlinico San Matteo, Pavia, Italy; b Servier Medical Affairs, Suresnes, France; c Soladis Clinical Studies, Roubaix, France; d Soladis, Lyon, France; e Department of Medical-Surgical Sciences and Biotechnologies, Sapienza University of Rome, Latina, Italy; f IRCCS Maria Cecilia Hospital, GVM Care & Research, Cotignola, Italy

**Keywords:** Cardiovascular disease, Meta-analysis, Mortality, Varicose veins, Venous insufficiency, chronic

## Abstract

We aimed to gather and summarize evidence on the potential links between chronic venous disease (CVeD), cardiovascular events and mortality in adults.

A systematic search of the PubMed database was conducted in accordance with PRISMA guidelines in October 2025. Observational studies or registries were eligible. Adults diagnosed with CVeD were compared with controls free of CVeD or varicose veins. Primary outcomes were cardiovascular events (angina, atrial fibrillation, chronic heart failure, coronary artery disease, deep vein thrombosis, stroke, myocardial infarction, peripheral artery disease, pulmonary embolism, transient ischaemic attack, composite of MACE) and all-cause mortality. Secondary outcomes focused on coronary artery and heart disease. Random-effects meta-analyses of time-to-event outcomes determined pooled hazard ratios (HRs) and 95% confidence intervals (CIs). The *I*^*2*^ statistic assessed heterogeneity.

Thirteen studies (6.4 million participants; 342,110 with CVeD) were included. Meta-analysis of nine studies (280,252 participants with CVeD) reported that CVeD was associated with a significantly increased risk of cardiovascular events (HR 1.5; 95% CI 1.1, 2.0; *p* = 0.006). Sensitivity analyses yielded consistent results. CVeD was also associated with an increased risk of all-cause mortality (five studies; 16,749 participants; HR 1.3; 95% CI 1.2, 1.6; *p* < 0.001) and of coronary artery and heart disease (six studies; 38,331 participants; HR 1.3; 95% CI 1.1, 1.6; *p* = 0.006). Heterogeneity was high (*I*^*2*^ 71%–95%).

Patients with CVeD appear to be at increased risk of cardiovascular events and all-cause mortality, particularly those with severe disease. Based on these data, the incremental value of including CVeD in cardiovascular risk assessments should be evaluated prospectively.

## Highlights

• We assessed the links between chronic venous disease and cardiovascular disease.
• This meta-analysis included 13 studies with over 6.4 million participants.
• Chronic venous disease was associated with a higher cardiovascular risk (HR 1.5).
• In advanced stages (CEAP C3-C6), all-cause mortality increased (HR 1.3).
• Chronic venous disease may be considered in cardiovascular risk assessments.

## Graphical abstract

**Figure:**
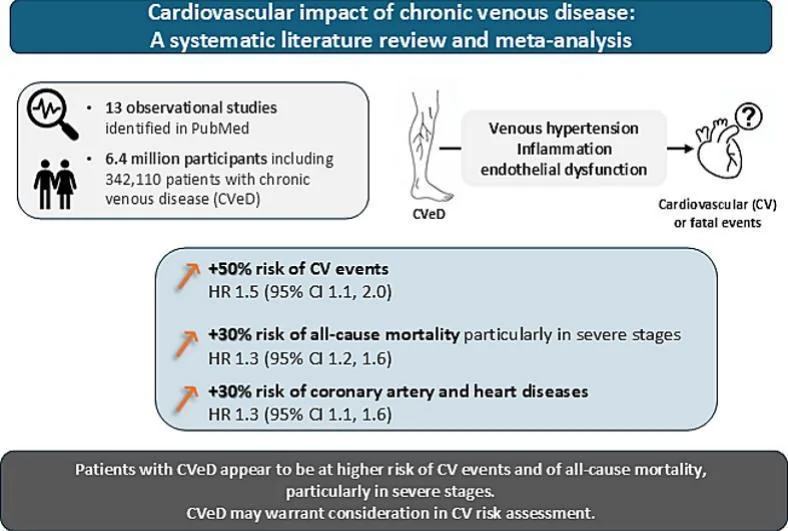

## 1 Introduction

Chronic venous disease (CVeD), a prevalent condition affecting a significant portion of the population, is characterized by the impaired return of blood from the legs to the heart due to malfunctioning venous valves [1]. This condition encompasses a spectrum of manifestations, from reticular veins and telangiectasias to more severe forms, such as chronic venous insufficiency (CVI) with oedema, dermal changes and ulceration [2], [3]. Varicose veins, the most common clinical sign of CVeD, are present in approximately 25% of affected individuals and are particularly prevalent in females, the elderly and those with a higher body mass index or a family history of the condition [1], [4], [5], [6], [7], [8]. In a 13-year follow-up study, almost half of patients with trunk varicose veins or CVI showed disease progression, while one-third of those with varicose veins developed skin changes increasing their risk of ulceration [9].

The morbidity burden associated with CVeD, including impaired quality of life comparable to that experienced with other chronic diseases, such as congestive heart failure and chronic lung disease, underscores its significant impact on patient well-being [10], [11], [12].

Several studies have suggested that CVeD and cardiovascular disease (CVD) share pathophysiological mechanisms, including inflammation and endothelial dysfunction, and common risk factors, such as diabetes mellitus, hypertension, obesity and smoking [8], [13], [14], [15], [16], [17]. These epidemiological investigations also demonstrated that patients with CVeD have an increased risk of major adverse cardiovascular events (MACE) and mortality [8], [13], [14], [15], [16], [17]. Consequently, this systematic literature review and meta-analysis was undertaken to comprehensively gather and analyse existing evidence exploring these potential links between CVeD and cardiovascular or fatal events.

## 2 Methods

### 2.1 Study design

This systematic review and meta-analysis was designed, conducted and reported in accordance with the PRISMA 2020 guidelines [18]. Because all included studies were observational, we also followed the MOOSE reporting recommendations [19]. The review protocol was registered in PROSPERO (CRD420251015163).

Studies included in the analysis comprised any retrospective or prospective studies (including cross-sectional, longitudinal observational studies or registries, irrespective of the duration of outcome follow-up) reporting cardiovascular data or mortality as study outcomes in patients diagnosed with CVeD. The study population consisted of adult patients diagnosed with any type of CVeD, regardless of the method of diagnosis and at any disease stage. Patients were included irrespective of whether they were receiving appropriate treatment for CVeD, including conservative or procedural treatments. The control group consisted of patients free of CVeD or varicose veins (i.e., Clinical, Etiological, Anatomical and Pathophysiological [CEAP] class C0–C1). The primary outcomes were the occurrence of any new cardiovascular events (including angina, atrial fibrillation [AF], chronic heart failure [HF], coronary artery disease [CAD], deep vein thrombosis [DVT], ischaemic or haemorrhagic stroke, myocardial infarction [MI], peripheral artery disease [PAD], pulmonary embolism [PE], transient ischaemic attack or a composite of MACE) and all-cause mortality. Patients could have a history of cardiovascular events but were free of the outcome of interest at the time they were included in the study. For example, a study evaluating the occurrence of AF in patients with CVeD may have included patients with previous MI or PAD.

Secondary outcomes comprised the occurrence of coronary artery and heart disease events, consisting of acute coronary syndrome (ACS), AF and HF.

### 2.2 Search strategy

A systematic literature search was performed, with no language restrictions, of the PubMed (MEDLINE) database on 31 October 2025 to identify articles published from 1 January 1960 to 31 October 2025. Full-text articles and conference abstracts were considered eligible. Reviews, systematic reviews, meta-analyses, study protocols, preclinical research, case reports, letters, notes and editorial publications were excluded from consideration. Additionally, any data that had not been formally published were also considered ineligible. The search strategy was developed in collaboration with a research librarian (AM). Searches were subsequently carried out by VBG and AM using the strategy outlined in Supplementary Table S1.

### 2.3 Study selection criteria

The titles and abstracts of the retrieved records were screened by VBG and MBO to identify relevant studies for further analysis and to exclude those that were clearly non-compliant or ineligible. After record selection, full texts were obtained. In addition, a manual search of the reference lists of the selected papers was conducted to identify other potentially eligible articles.

### 2.4 Data extraction and outcomes

Two independent reviewers (VBG and MBO) entered relevant data from the included articles on dedicated data collection forms. Any discrepancies between the reviewers were resolved by consensus. The information collected included the title, authors' name, year of publication, study region, total number of patients, inclusion and exclusion criteria, study duration, patients' demographic and baseline characteristics and relevant outcomes.

### 2.5 Quality assessment

The quality of the included studies was assessed using the Newcastle-Ottawa Scale [20], [21]. This scale is used to evaluate non-randomised studies in meta-analyses and includes selection, comparability and outcome categories. Scoring is rated as follows: 7–9 points are considered as ‘high’, 4–6 as ‘moderate’ and 0–3 as ‘low’ quality.

### 2.6 Assessment of publication bias

Publication bias was explored qualitatively using visual inspection of funnel plots. Formal statistical tests for funnel plot asymmetry were not performed because fewer than 10 studies contributed to each outcome-specific meta-analysis [22].

### 2.7 Statistical analyses

Descriptive summaries were provided for patient demographics including mean and standard deviation (SD). Percentages were calculated on the number of participants with available data unless otherwise specified.

For the primary outcomes (i.e., new cardiovascular events and all-cause mortality), adjusted hazard ratios (HRs) reported by each study were pooled using a three-level random-effects meta-analysis. Because several studies reported multiple clinically distinct cardiovascular outcomes from the same cohort, all eligible outcome-specific estimates were retained and analysed within a three-level framework that accounted for their statistical dependence, rather than selecting a single estimate per study [23], [24], [25]. In this framework, level 1 represents the sampling variance of each effect size, level 2 the variability among multiple outcome-specific effect sizes reported within the same study, and level 3 the heterogeneity between studies. Random effects were specified to account for heterogeneity both within and between studies, and parameters were estimated using restricted maximum likelihood. No fixed correlation coefficient between outcomes was assumed or specified. HRs were pooled using a generic inverse-variance approach, with weights adjusted to account for correlations between outcomes within the same study. The analysis was implemented using the ‘rma.mv function' in the metafor R package, allowing the model to estimate appropriate variance components based on the data.

Across the included studies, adjusted HRs were derived from multivariable Cox models or propensity score-matched cohorts, with covariate adjustment typically incorporating demographic factors (age, sex), cardiovascular risk factors (hypertension, diabetes, dyslipidaemia, obesity), major comorbidities and when available, lifestyle habits (e.g., smoking or alcohol use). Several studies were additionally adjusted for healthcare use proxies, socioeconomic indicators or medications, such that the pooled estimates reflected associations adjusted for the several major measured confounders.

To maximize the use of available data, incidence rate ratios (IRRs) were approximated as hazard ratios (HRs), consistent with methodological approaches suggesting that IRRs may reasonably approximate HRs when hazards are relatively stable over time and event rates are low. The logarithm of the IRR was used as an approximation of the log-HR (lnHR ≈ lnIRR) [26], [27], [28], [29], [30]. Sensitivity analyses restricted to studies reporting HRs were conducted to assess the robustness of this approach.

As an additional sensitivity analysis, the analysis of the primary outcome was repeated after removing outcome-specific estimates for venous thromboembolic outcomes (DVT and PE) to evaluate whether the overall association between CVeD and cardiovascular events was disproportionately influenced by these events.

Heterogeneity was assessed using the *I*^2^ statistic, Cochran's *Q* test, and graphically using the forest plot. Overall, results were provided as a pooled estimate of the HRs for the association of CVeD with any cardiovascular event, combining all reported outcomes across studies, along with the corresponding 95% confidence interval (CI) and *p*-value (a two-sided *p*-value <0.05 was considered statistically significant).

Regarding secondary outcomes, estimates of the HRs (plus 95% CIs and *p*-values) of the associations of CVeD with coronary artery and heart disease were provided following the same methods applied for the primary outcomes. All statistical analyses were conducted using R software (version 4.4.0, www.r-project.org).

## 3 Results

A total of 13 studies were included in the systematic review (Table 1, Fig. 1) [8], [15], [17], [31], [32], [33], [34], [35], [36], [37], [38], [39]. These studies were conducted in Europe (seven studies) and Asia (six studies). Twelve studies were cohorts, and one was a case-control study [39], with most being retrospective and two prospective [15], [35]. Altogether, the included studies comprised nearly 6.4 million participants (*N* = 6,391,569), of whom 342,110 had CVeD. Notably, four studies from Taiwan and South Korea based on national health databases contributed most of the participants, representing 81.4% of the total sample (5,202,712 of 6,391,569) and 78.4% of patients with CVeD (268,335 of 342,110) [8], [31], [32], [33].

**Table 1:** Summary of studies included in the systematic review.

Study	Country	Study design	Study population	Duration of follow-up; years^a^	Total patients studied (N)	Total patients with CVeD (n)	Control group without CVeD (n)	Principal findings
Chang 2018 [8]	Taiwan	r, co, claims	Pts aged ≥20 y with VV	7.3–7.8	1,064,920	212,984	851,936^b^	↑ risk of DVT (IR 6.55 vs 1.08 per 1000 PY), PAD (IR 10.73 vs 5.20 per 1000 PY), PE (IR 0.48 vs 0.24 per 1000 PY)
Choi 2024 [31]	Korea	r, co, claims	Pts aged ≥40 y with VV	10.06 (IQR 9.43–10.44)	2,680,971	24,557	2,656,414	Significantly ↑ risk of AF (IR 3.95 vs 3.04 per 1000 PY; *p* < 0.001)
Hsieh 2025 [32]	Taiwan	r, co, claims	Pts aged ≥20 y with or without VV	18	1,428,926	25,215	1,403,711^c^	The adjusted hazard ratio (aHR) for overall stroke in the VV group was 1.16 (95%CI, 1.11–1.21)
Hu 2022 [33]	Taiwan	r, co, claims	Adults with or without VV	13*	27,895	5579	22,316	↑ prevalence (p < 0.001) of CAD, HF, PAD, stroke, VTE; ↑ risk of AF (IR 4.82 vs 3.47 per 1000 PY; p < 0.001)
Kim 2025 [34]	Korea	r, co	Adults with or without VV	13.33 (IQR 10.40–16.26)	390,436	5008	385,428	Significantly ↑ risk of HF (aHR 1.174; p < 0.001)
Kreft 2020 [35]	Germany	pr, co	Pts aged ≥50 y with or without VV	≤12*	237,411	4673	–	VLU ↑ mortality risk (age-standardised mortality rate 70.2 per 1000 PY, vs 28.0 per 1000 PY for total study population)
Mäkivaara 2007 [36]	Finland	r, co	Three pt. co (aged 40, 50, or 60 y) with or without VV	5*	4903	1567	3065	VV significantly ↑ incidence of arterial disease (IOR 2.0) but not HT (IOR 1.0)
Mäkivaara 2009 [37]	Finland	r, co	Three pt. co (aged 40, 50, or 60 y) with or without VV	5*	4903	1567	3065	↑ prevalence of CHF (OR 1.6)^d^ Significantly ↑ incidence of CHF (IDR 1.9)
Prochaska 2021(a) [15]	Germany	pr, co	An analysis sample providing information on the clinical status of CVI	6.4 ± 1.6*^,^^e^	10,664	9758	906	Advanced CVI (C4–C6 vs C0) significantly associated with cardiovascular disease (PR 1.10; *p* = 0.003), PAD (PR 1.13; *p* = 0.006), and VTE (PR 1.18; *p* < 0.001) CVI (C3–C6 vs C0–C2) significantly associated with increased risk of all-cause mortality (*p* < 0.0001)
Prochaska 2021(b) [15]	Germany	pr, co	External validation (MyoVasc) co	3.2 ± 1.1*^,^^e^	2423^f^	2423	–	CVI independent predictor of all-cause mortality (HR 1.51; *p* = 0.009)
Rahman 2011 [38]	UK	r, co	Pts with or without VV or haemorrhoids	35*	508,257	43,724	464,533	VV significantly ↓ risk of CHD (aRR 0.91) and MI (aRR 0.89) VV significantly ↑ risk of DVT (aRR 1.20); significantly ↑ risk of HF <5 years after hospital admission (aRR 1.18)
Salenius 2021 [39]	Finland	r, cc	Pts aged ≥18 y with or without chronic ulcers	≤10 (IQR 3–17)	12,377	1978	10,399	Significantly ↑ mortality risk (HR 1.49) in patients with VLU
Wu 2020 [17]	Taiwan	r, co	Pts with or without VV	17*	22,386	4644	17,742	Significantly ↑ risks of MACE (aHR 2.05; *p* < 0.0001) and all-cause mortality (aHR 1.37; p < 0.0001)

↑, increase(d); ↓, decrease(d); AF, atrial fibrillation; aHR, adjusted hazard ratio; aOR, adjusted odds ratio; aRR, adjusted rate ratio; CAD, coronary artery disease; cc, case-control; CEAP, Clinical, Etiology, Anatomy, and Pathophysiology; CHD, coronary heart disease; CHF, congestive heart failure; co, cohort; cs, cross-sectional; CVeD, chronic venous disease; CVI, chronic venous insufficiency; DVT, deep vein thrombosis; *epi*, epidemiological; ETA, endovenous thermal ablation; HF, heart failure; HR, hazard ratio; HT, hypertension; IC, intermittent claudication; IDR, incidence density ratio; IHD, ischaemic heart disease; IOR, incidence odds ratio; IQR, interquartile range; IR, incidence rate; IVS, iliac vein stenting; MACE, major adverse cardiovascular events; mc, multicentre; MI, myocardial infarction; NS, no(t) significant; OR, odds ratio; PAD, peripheral artery disease; PE, pulmonary embolism; pH, pulmonary hypertension; pr, prospective; PR, prevalence ratio; PS, propensity score; PY, person-years; r, retrospective; VLU, venous leg ulcers; VTE, venous thromboembolism; VV, varicose veins; y, years.

a Median value listed, unless indicated by an asterisk* (= full study duration).

b Control group matched on age, sex and calendar year.

c Control group matched on age, sex, relevant comorbidities and medications.

d NS after adjustment for age and sex.

e Mean ± standard deviation.

f MyoVasc study cohort (ClinicalTrials.gov identifier: NCT04064450).

**Fig. 1:**
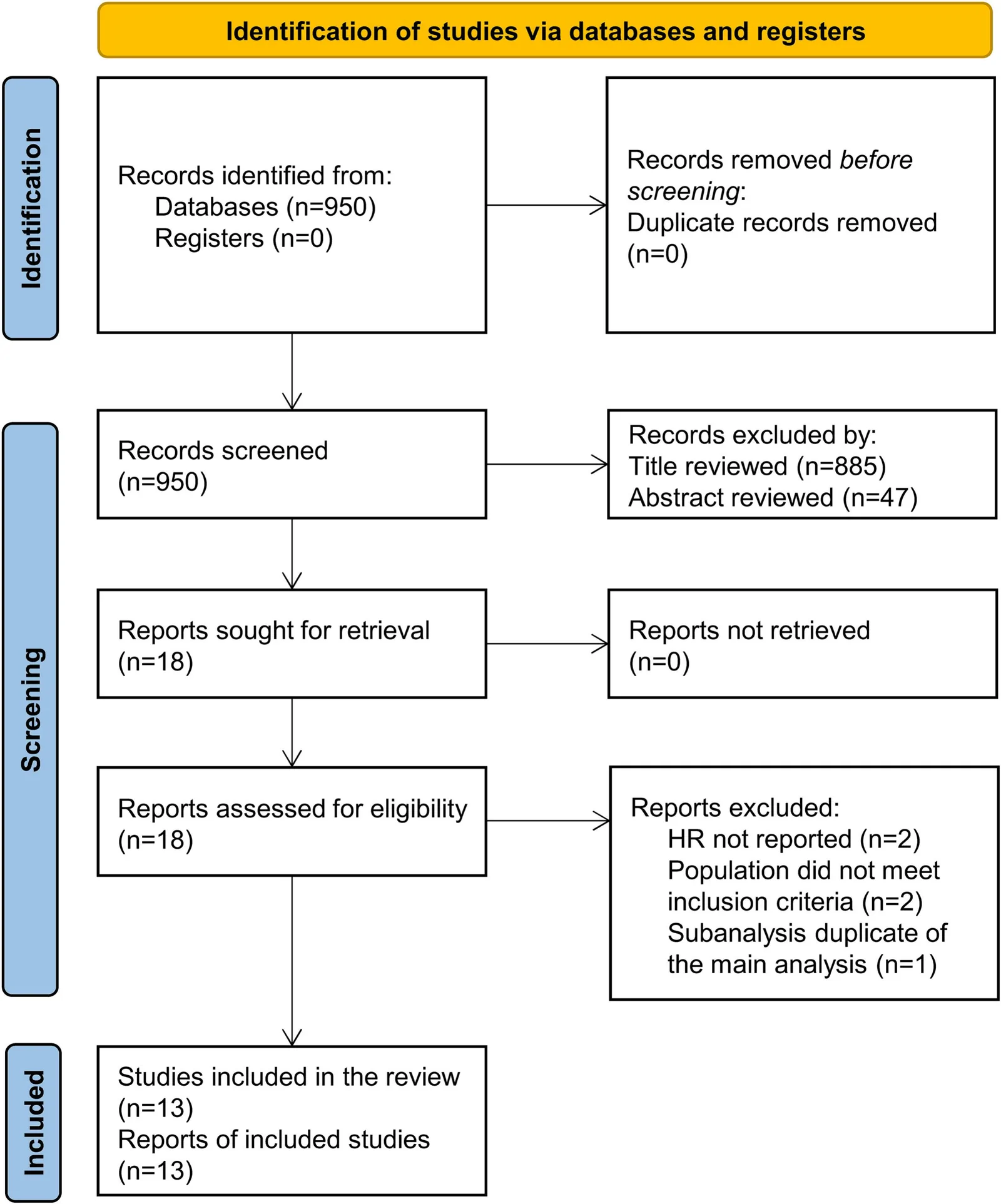
.PRISMA flow chart. HR, hazard ratio.

Overall, in the total sample of participants who had baseline demographic data available, mean ± SD age was 50.9 ± 14.7 years (*n* = 4,315,885), 54.5% of participants were female (*n* = 4,615,811) and mean ± SD body mass index was 23.8 ± 3.2 kg/m^2^ (*n* = 3,071,407). Duration of follow-up varied from 3 to 17 years.

Regarding CVeD severity, most studies included patients with varicose veins but did not specify their CEAP clinical class. Prochaska and colleagues enrolled patients across the full CEAP spectrum (CEAP C0–C6) and conducted subgroup analyses specifically in patients with more advanced disease (C3–C6) [15]. Additionally, two studies reported outcomes in individuals with venous ulcers, corresponding to the most severe CEAP clinical class (C6) [35], [39].

Among participants with available data, the prevalence of cardiovascular risk factors was 25.2% for hypertension (1,083,402/4,291,076; six studies [8], [15], [31], [32], [33], [34]), 27.4% for obesity (2926/10,664; one study [15]), 18.2% for hyperlipidaemia (780,182/4,280,412; five studies [8], [31], [32], [33], [34]), 10.5% for diabetes (448,581/4,291,076; six studies [8], [15], [31], [32], [33], [34]) and smoking was reported in 23.3% (718,366/3,082,071; three studies [15], [31], [34]). Previous cardiovascular events included ischemic heart disease (14.5% [154,167/1,064,920]; one study [8]), coronary artery/heart disease (18.5% [28,621/154,749]; three studies [15], [32], [33]), PAD (4.9% [1877/38,559]; two studies [15], [33]), and venous thrombo-embolism (2.4% [932/38,559]; two studies [15], [33]). Rates of previous AF, HF, stroke and MI did not exceed 2%.

Based on the Newcastle-Ottawa Scale rating, 11 studies were rated as a total score of >7 [8], [15], [17], [31], [32], [33], [34], [35], [38], [39], indicating a high quality and two studies as a score of 6 [36], [37], indicating a moderate quality (Supplementary Fig. S1).

### 3.1 Effects on cardiovascular events.

Nine studies on cardiovascular events (280,252 participants with CVeD) reported relevant data for HRs to be compared; six of those studies reported HRs [8], [17], [31], [32], [33], [34] and three reported IRRs approximated as HRs [36], [37], [38]. The meta-analysis showed that CVeD was associated with an increased risk of cardiovascular events (HR 1.5; 95% CI 1.1, 2.0; *p* = 0.006; Fig. 2, Supplementary Table S2). Heterogeneity between studies was high (*Q* = 7427.17, *p* < 0.01; *I*^2^ = 95%) and visual inspection of the funnel plot did not suggest publication bias (Supplementary Fig. S2). Sensitivity analysis restricted to studies reporting HRs revealed that the overall risk for cardiovascular events remained statistically significant (HR 1.6; 95% CI 1.1, 2.4; *p* = 0.010; Supplementary Fig. S3). In an additional sensitivity analysis excluding outcome-specific estimates of DVT and PE, CVeD remained significantly associated with cardiovascular events (HR 1.4; 95% CI 1.1, 1.7; *p* = 0.002; Supplementary Fig. S4), indicating that the observed association was not primarily driven by venous thromboembolic outcomes.

**Fig. 2:**
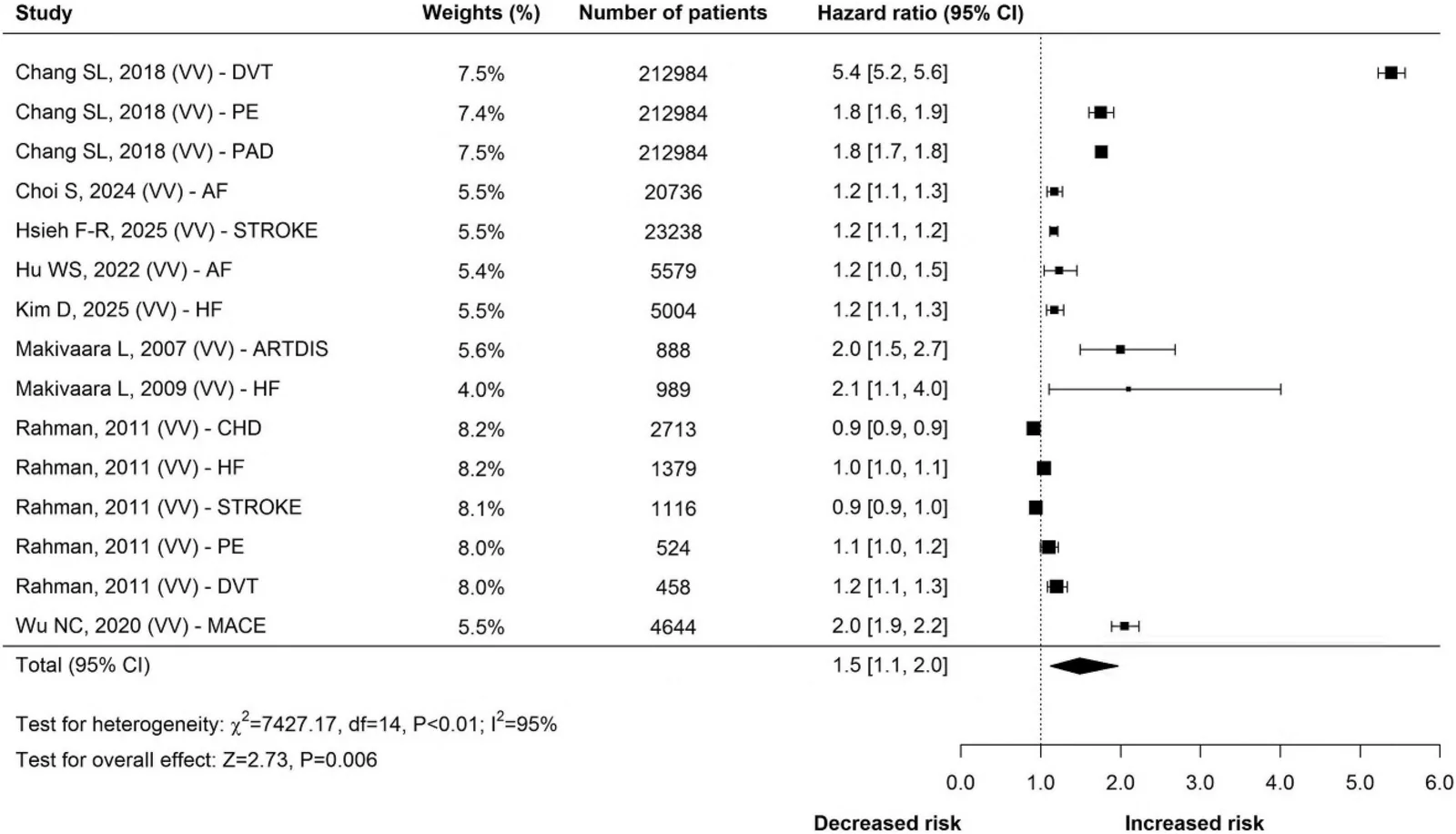
Forest plot showing the hazard ratios^a^ of the association between chronic venous disease and cardiovascular events (9 studies; 15 outcomes) [8], [17], [31], [32], [33], [34], [36], [37], [38]. ^a^In three studies, values for IRRs were approximated as hazard ratios [36], [37], [38]. AF, atrial fibrillation; ARTDIS, arterial disease; CHD, coronary heart disease; CI, confidence interval; DVT, deep vein thrombosis; HF, heart failure; IRR, incidence rate ratio; MACE, major adverse cardiovascular events; PAD, peripheral artery disease; PE, pulmonary embolism; VV, varicose veins.

### 3.2 Effects on all-cause mortality.

Five studies on all-cause mortality (16,749 participants with CVeD) reported relevant data for HRs to be compared [15], [17], [35], [39]. Among them, an independent cohort study [MyoVasc, NCT04064450] [15] including patients with HF was used as an external validation cohort showing that presence of CVI independently predict all-cause mortality, consistent with findings of the German study [15]. The pooled meta-analysis revealed that CVeD was associated with an increased risk of all-cause mortality (HR 1.3; 95% CI 1.2, 1.6; *p* < 0.001; Fig. 3, Supplementary Table S3). Heterogeneity was substantial (*Q* = 78.91, *p* < 0.01; *I*^2^ = 90%) and the funnel plot did not indicate publication bias (Supplementary Fig. S5).

**Fig. 3:**
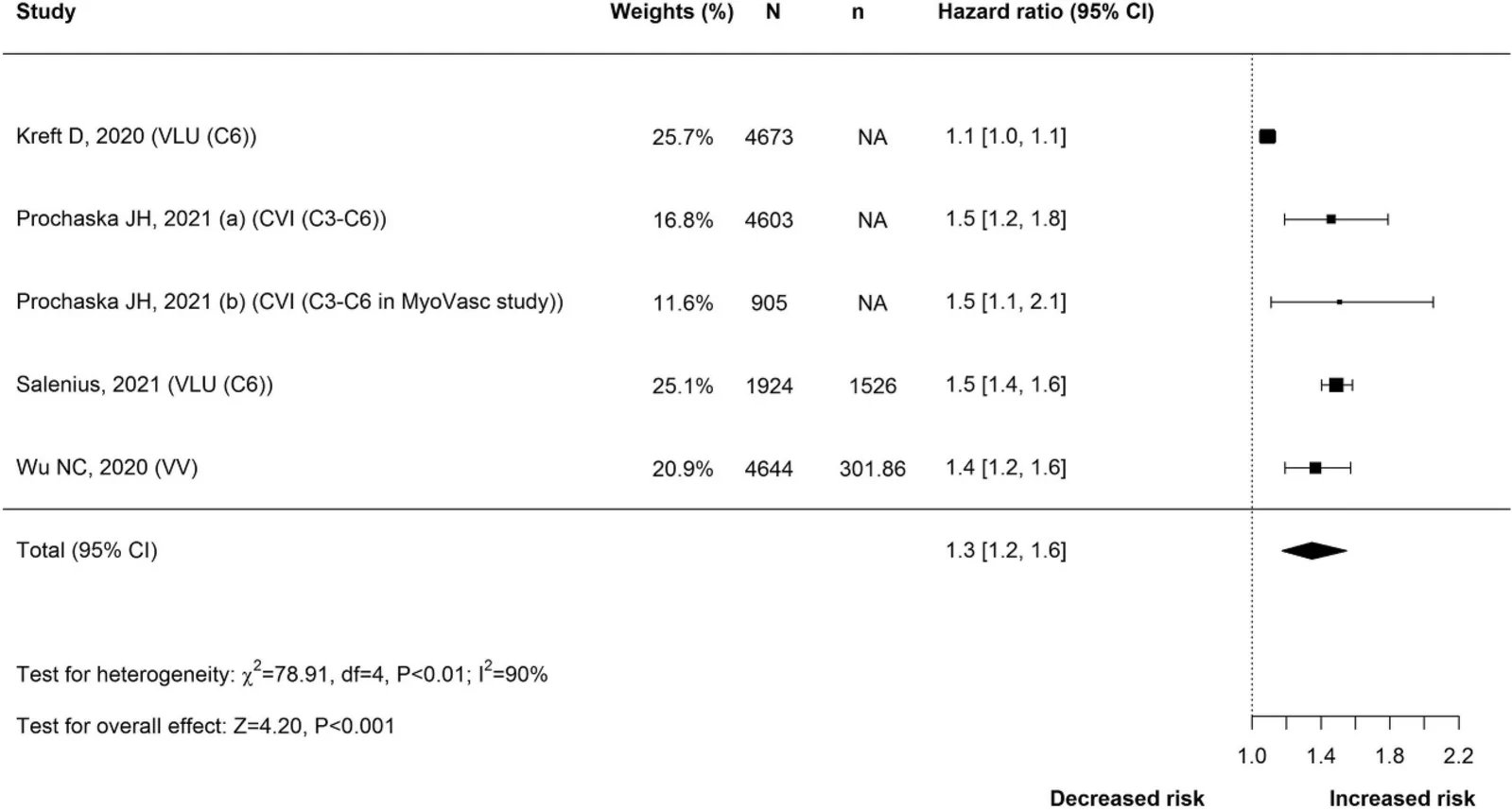
Forest plot showing the hazard ratios for the association between chronic venous disease and all-cause mortality (5 studies; 5 outcomes) [15], [17], [35], [39]. ‘N' means the number of patients with data available; ‘n' means the number of deaths reported. CI, confidence interval; CVI, chronic venous insufficiency; VLU, venous leg ulcer; VV, varicose veins.

### 3.3 Effects on coronary artery and heart disease.

Six studies involving 38,331 participants with CVeD reported HRs for coronary artery and heart disease outcomes [17], [31], [33], [34], [37], [38]. The meta-analysis revealed that CVeD was associated with an increased risk of coronary artery and heart disease (HR 1.3; 95% CI 1.1, 1.6; *p* = 0.006; Supplementary Fig. S6 and Supplementary Table S4). Heterogeneity was substantial (*Q* = 112.63, *p* < 0.01; *I*^2^ = 71%) and no asymmetry was observed on visual inspection of the funnel plot (Supplementary Fig. S7).

## 4 Discussion

Our systematic literature review and meta-analysis investigated the association between CVeD and cardiovascular and fatal events in adults. Given the fragmented nature of the available evidence, encompassing cardiac, arterial, and venous thromboembolic outcomes, a broad composite endpoint was selected to capture the overall vascular burden associated with CVeD across the vascular continuum. The inclusion of venous thromboembolic events, such as deep vein thrombosis and pulmonary embolism, alongside cardiac and arterial outcomes, was supported by evidence that CVeD shares pathophysiological mechanisms with both arterial and venous thromboembolic diseases. These mechanisms include systemic inflammation, endothelial dysfunction, vascular remodelling, and activation of prothrombotic pathways [14], [15], [40]. Analysing data from 13 cohort studies comprising more than 6.4 million participants, this meta-analysis found that CVeD was significantly associated with a 50% higher risk of cardiovascular events, a 30% higher risk of coronary artery and heart disease events and a 30% higher risk of all-cause mortality. Although the highest individual risk estimates were observed for deep vein thrombosis and pulmonary embolism, the association with cardiovascular events remained significant after excluding these outcome-specific estimates. The significant association observed in the prespecified analysis restricted to coronary artery and heart disease further indicated that the overall findings were not solely driven by venous thromboembolic outcomes.

These findings are consistent with a recent systematic review which analysed data from 20 studies including almost 400,000 individuals and reported a significant association between CVeD and cardiovascular outcomes, especially in moderate-to-severe cases [41]. The Framingham Heart Study was the first to investigate the association between varicose veins and atherosclerotic CVD, reporting an elevated risk particularly among women [42]. However, this association was not independent of confounding factors, such as obesity and hypertension, which are prevalent in both conditions. Thirty years later, Prochaska et al. demonstrated that chronic venous insufficiency (CEAP C3-C6) was associated with a greater burden of cardiovascular disease and higher mortality after adjustment for established risk factors [15]. In this study, disease severity was strongly and progressively associated with all-cause mortality, with moderate (C3) and advanced stages exhibiting higher risks. Wu et al. [17] similarly reported progressively increasing risks of major adverse cardiovascular events and mortality with increasing varicose vein severity, while Choi et al. [31] observed an increased risk of atrial fibrillation in both mild and severe varicose veins compared with matched controls, with numerically higher estimates in patients with severe disease. Collectively, these observations suggest that cardiovascular risk may increase with CVeD severity.

In the present analysis, the pooled mortality estimate was predominantly informed by studies including patients with chronic venous insufficiency (CEAP C3–C6) [15] or venous ulceration (C6) [35], [39], who may have had a greater burden of comorbidities and established cardiovascular disease. Despite adjustment for major confounders in the original studies, residual confounding cannot be excluded, and the observed association with mortality may partly reflect the broader vascular and cardiometabolic risk profile of patients rather than a direct effect of venous disease itself. Therefore, the mortality findings should be interpreted cautiously.

CVeD encompasses vascular disorders resulting in significant local and systemic consequences. Various mechanisms have been proposed to explain the link between CVeD, CVD and mortality. Indeed, alterations in venous compliance, as observed in CVeD, may affect venous return and exert downstream effects on arterial remodelling and cardiac function [43], [44]. For example, patients with varicose veins exhibit altered cardiac Doppler parameters [45], [46], and disease severity from CEAP class C3 onward correlates with cardiac haemodynamic changes [47], [48]. Furthermore, elevated preoperative BNP levels have been reported in 60.7% of patients with varicose veins, and interventions targeting saphenous vein insufficiency have been shown to significantly reduce these levels [49]. Additionally, therapeutic strategies for CVI, such as radiofrequency ablation or surgical intervention, have been shown to improve cardiac function [50], [51].

Emerging evidence suggests a shared pathophysiology between venous and arterial diseases, driven by systemic inflammation, oxidative stress, and endothelial dysfunction [13], [14], [15], [40]. In CVeD, localized vascular inflammation due to venous hypertension and endothelial activation is also accompanied by elevated circulating pro-inflammatory cytokines, chemokines and C-reactive protein [4], [52], [53], [54]. Endothelial dysfunction is a precursor of adverse cardiovascular outcomes [13], [55] and also emerges as a pivotal contributor to thrombogenesis across the venous and arterial systems [56], [57], [58]. Furthermore, lower-limb oedema associated with CVI may trigger inflammation that elevates the risk of deep vein thrombosis [59]. These overlapping mechanisms provide biological plausibility for shared disease pathways.

In addition to the overlap of numerous genes in the pathophysiology of both CVeD and arterial disease [5], [14], [60], [61], [62], the causality between CVeD and CVD was assessed using Mendelian randomization and genome-wide association studies analyses. These latter analyses reveal genetic overlaps between varicose veins and arterial diseases like PAD and aortic aneurysm [63]. Mendelian randomization analyses suggest a causal, unidirectional relationship between varicose veins and HF [64], and between varicose veins and AF [65], but not with hypertension, coronary artery disease or stroke [66]. These genetic analyses used to evaluate potential causal relationships between exposures and clinical outcomes reinforce the link between CVeD and cardiovascular risk.

### 4.1 Limitations and strengths.

This meta-analysis is the first to specifically assess the association between all-cause mortality and coronary and heart disease with CVeD. While this work identified significant associations, several limitations should be noted. These include substantial heterogeneity likely reflecting differences in disease severity, geographic regions, study design and follow-up duration. Most studies identified patients based on the presence of varicose veins without providing detailed CEAP classifications, while four large studies from Taiwan and South Korea accounted for more than 80% of the overall study population. In addition, the included studies were predominantly observational cohort studies, with only one case-control study [39], and follow-up duration varied considerably across studies, ranging from approximately 3 to 17 years. Although investigation of heterogeneity through subgroup analyses or meta-regression would have been of interest, key study-level characteristics were incompletely reported, and the limited number of studies contributing to each outcome provided insufficient statistical power for robust and interpretable analyses.

Furthermore, treatment details, particularly regarding venous interventions that may be associated with DVT [67], were reported inconsistently across the studies included in our review. However, evidence of patients undergoing endovenous thermal ablation for varicose veins revealed that this procedure significantly reduces the risk of thromboembolism and PAD compared to no treatment [68]. Other studies have reported either no effect or possible benefits of venous surgery on AF and HF occurrence [31], [34]. Taken together, this indicate that the inclusion of patients who had undergone venous procedural interventions may not artificially inflate the estimated cardiovascular risk.

Although IRRs were approximated as HRs, residual approximation error cannot be excluded. However, sensitivity analysis restricted to studies reporting HRs yielded similar results, suggesting that the approximation did not substantially affect the pooled estimates.

The quality of the observational studies included in our meta-analysis was assessed using the Newcastle–Ottawa Scale, a straightforward and widely used tool for evaluating observational studies [20]. However, several items have uncertain validity and may lead to subjective or inconsistent quality ratings, which can affect how differences between studies are interpreted [21].

Regarding the assessment of publication bias, interpretation of the funnel plots requires caution because of the small number of included studies (<10 for each outcome) and that each point represents an outcome-specific effect estimate rather than an independent study. As a result, the funnel plots reflected a mixture of within-study and between-study variability. Egger's regression test was not performed because fewer than 10 studies contributed to each outcome-specific meta-analysis; according to Cochrane Handbook recommendations, such tests have limited power and are not recommended under these circumstances [22]. Despite these methodological limitations, no obvious asymmetry was visually apparent for either the primary or secondary outcomes.

A strength of the meta-analysis is that all included studies applied propensity-score matching or adjusted HRs, accounting for several major measured confounders. Nevertheless, residual confounding and unmeasured differences between patient populations cannot be excluded and additional prospective studies are needed to better characterize the independent contribution of CVeD to mortality risk.

### 4.2 Clinical Implications and Conclusions.

Overall, this meta-analysis contributes to the existing literature by consolidating evidence of the association between CVeD and major cardiovascular outcomes and all-cause mortality. Available evidence suggests that this association may be stronger in patients with more advanced disease. From an epidemiological perspective, CVeD could be considered as a risk modifier, since it commonly coexists with major cardiometabolic conditions, such as obesity, sedentary lifestyle, as well as hypertension. However, emerging genetic and Mendelian randomization studies suggest a directional causal relationship between CVeD and specific cardiovascular outcomes, particularly AF and HF.

Clinical signs, such as varicose veins, oedema, skin changes and venous ulcers, may reflect systemic vascular involvement rather than isolated venous pathology, suggesting the importance of recognizing their potential clinical relevance, which goes beyond aesthetic considerations. These highlight the importance of identifying CVeD patients with coexisting cardiometabolic conditions and comprehensively evaluate their cardiovascular risk [14], [15], [40], [69], [70]. This supports a more integrated approach to managing both venous disease and overall cardiovascular risk, which would require education and collaboration between specialists [40], [69], [70]. Further research is needed to clarify the causal relationship, to investigate the impact of varicose vein treatment on cardiovascular risk, and to evaluate interventions targeting shared pathways between CVeD and CVD among these patients with multiple comorbidities.

## Previous presentation

The results of the study were presented in the form of a poster at the European Society of Cardiology Preventive Cardiology Congress 2026, which took place from 23 to 25 April in Ljubljana, Slovenia.

## CRediT authorship contribution statement

**Leonardo De Luca:** Writing – review & editing, Methodology, Formal analysis, Conceptualization. **Vanessa Blanc-Guillemaud:** Writing – review & editing, Supervision, Methodology, Formal analysis, Conceptualization. **Marie-Blanche Onselaer:** Writing – review & editing, Supervision, Methodology, Formal analysis, Conceptualization. **Soumeya Serifou:** Writing – review & editing, Methodology, Formal analysis, Conceptualization. **Hurrem Pelin Yaltirik:** Writing – review & editing, Methodology, Formal analysis, Conceptualization. **Giuseppe Biondi-Zoccai:** Writing – review & editing, Methodology, Formal analysis, Conceptualization.

## Informed consent

Considering the nature of this systematic review and meta-analysis, informed consent was not required.

## Ethical approval

Considering the nature of this systematic review and meta-analysis, ethical approval was not required.

## Human and animal rights

This is a systematic review and meta-analysis of outcomes and did not involve direct human or animal participation.

## Funding

This work was supported by Servier. Soladis Clinical Studies (Roubaix, France) and Soladis (Lyon, France) received fees from Servier for the conduct of this work. The development of the manuscript and open access publishing fee were supported by Servier.

## Declaration of competing interest

The authors declare the following financial interests/personal relationships which may be considered as potential competing interests: Leonardo De Luca reports financial support, article publishing charges, travel, and writing assistance were provided by Servier. Vanessa Blanc-Guillemaud, Marie-Blanche Onselaer, Soumeya Serifou, Hurrem Pelin Yaltirik, Giuseppe Biondi-Zoccai report financial support, article publishing charges, and writing assistance were provided by Servier. Vanessa Blanc-Guillemaud and Hurrem Pelin Yaltirik report a relationship with Servier that includes: employment. Marie-Blanche Onselaer reports a relationship with Soladis Clinical Studies, France that includes: employment. Soumeya Serifou reports a relationship with Soladis, France that includes: employment. Giuseppe Biondi-Zoccai reports a relationship with Abiomed that includes: board membership, consulting or advisory, paid expert testimony, and speaking and lecture fees. Giuseppe Biondi-Zoccai reports a relationship with Advanced Nanotherapies that includes: board membership, consulting or advisory, paid expert testimony, and speaking and lecture fees. Giuseppe Biondi-Zoccai reports a relationship with Amarin that includes: board membership, consulting or advisory, paid expert testimony, and speaking and lecture fees. Giuseppe Biondi-Zoccai reports a relationship with AstraZeneca that includes: board membership, consulting or advisory, paid expert testimony, and speaking and lecture fees. Giuseppe Biondi-Zoccai reports a relationship with Balmed that includes: board membership, consulting or advisory, paid expert testimony, and speaking and lecture fees. Giuseppe Biondi-Zoccai reports a relationship with Cardionovum that includes: board membership, consulting or advisory, paid expert testimony, and speaking and lecture fees. Giuseppe Biondi-Zoccai reports a relationship with Cardionovum that includes: board membership, consulting or advisory, paid expert testimony, and speaking and lecture fees. Giuseppe Biondi-Zoccai reports a relationship with Cepton that includes: board membership, consulting or advisory, paid expert testimony, and speaking and lecture fees. Giuseppe Biondi-Zoccai reports a relationship with Cordis that includes: board membership, consulting or advisory, paid expert testimony, and speaking and lecture fees. Giuseppe Biondi-Zoccai reports a relationship with Crannmedical that includes: board membership, consulting or advisory, paid expert testimony, and speaking and lecture fees. Giuseppe Biondi-Zoccai reports a relationship with Endocore Lab that includes: board membership, consulting or advisory, paid expert testimony, and speaking and lecture fees. Giuseppe Biondi-Zoccai reports a relationship with Eukon that includes: board membership, consulting or advisory, paid expert testimony, and speaking and lecture fees. Giuseppe Biondi-Zoccai reports a relationship with Guidotti that includes: board membership, consulting or advisory, paid expert testimony, and speaking and lecture fees. Giuseppe Biondi-Zoccai reports a relationship with Innova HTS that includes: board membership, consulting or advisory, paid expert testimony, and speaking and lecture fees. Giuseppe Biondi-Zoccai reports a relationship with Innovheart that includes: board membership, consulting or advisory, paid expert testimony, and speaking and lecture fees. Giuseppe Biondi-Zoccai reports a relationship with Menarini that includes: board membership, consulting or advisory, paid expert testimony, and speaking and lecture fees. Giuseppe Biondi-Zoccai reports a relationship with Microport that includes: board membership, consulting or advisory, paid expert testimony, and speaking and lecture fees. Giuseppe Biondi-Zoccai reports a relationship with Opsens Medical that includes: board membership, consulting or advisory, paid expert testimony, and speaking and lecture fees. Giuseppe Biondi-Zoccai reports a relationship with Otsuka Medical Devices Europe that includes: board membership, consulting or advisory, paid expert testimony, and speaking and lecture fees. Giuseppe Biondi-Zoccai reports a relationship with Recor that includes: board membership, consulting or advisory, paid expert testimony, and speaking and lecture fees. Giuseppe Biondi-Zoccai reports a relationship with SciSpace that includes: board membership, consulting or advisory, paid expert testimony, and speaking and lecture fees. Giuseppe Biondi-Zoccai reports a relationship with Servier that includes: board membership, consulting or advisory, paid expert testimony, and speaking and lecture fees. Giuseppe Biondi-Zoccai reports a relationship with Synthesa that includes: board membership, consulting or advisory, paid expert testimony, and speaking and lecture fees. Giuseppe Biondi-Zoccai reports a relationship with Terumo and Translumina that includes: board membership, consulting or advisory, paid expert testimony, and speaking and lecture fees. Giuseppe Biondi-Zoccai is also an inventor of the patents “IT202200001988A1: Balloon anchorable vascular catheter” and “IT202300010854A1: Device for traction and bandaging of the left atrial appendage.” Leonardo De Luca reports a relationship with Servier that includes: travel reimbursement. If there are other authors, they declare that they have no known competing financial interests or personal relationships that could have appeared to influence the work reported in this paper.

## Data Availability

The datasets used and/or analysed during the current systematic review and meta-analysis are available from the corresponding author on reasonable request.
