# Supplementary material for: Cardiovascular impact of chronic venous disease: A systematic review and meta-analysis
Source: Int J Cardiol Heart Vasc. 2026 Oct 1;67:102024. doi: 10.1016/j.ijcha.2026.102024 (PMC13656867; doi:10.1016/j.ijcha.2026.102024)
Supplement: Supplementary file 1 [file mmc1.docx]

# Supplementary Material

**Cardiovascular impact of chronic venous disease: a systematic review and meta-analysis**

**Authors:** Leonardo De Luca^1^, Vanessa Blanc-Guillemaud^2^, Marie-Blanche Onselaer^3^, Soumeya Serifou^4^, Hurrem Pelin Yaltirik^2^, Giuseppe Biondi-Zoccai^5,6^

**Affiliations:** ^1^Division of Cardiology, Fondazione IRCCS Policlinico San Matteo, Pavia, Italy; ^2^Servier Medical Affairs, Suresnes, France; ^3^Soladis Clinical Studies, Roubaix, France; ^4^Soladis, Lyon, France; ^5^Department of Medical-Surgical Sciences and Biotechnologies, Sapienza University of Rome, Latina, Italy; ^6^ IRCCS Maria Cecilia Hospital, GVM Care & Research, Cotignola, Italy

**Short running head**: Cardiovascular impact of venous disease: a meta-analysis

**Funding:** This study was funded by Servier, France.

**Corresponding author:** Prof. Leonardo De Luca, Director, Cardiology Division, Fondazione IRCCS Policlinico San Matteo, Viale Camillo Golgi 19, 27100 Pavia, Italy. Tel: 0382.503158. Email: [l.deluca@smatteo.pv.it](mailto:l.deluca@smatteo.pv.it)

## Table S1.

Search strategy

The search terms included:

((("venous insufficiency"[MeSH Terms]) OR ("varicose veins"[MeSH Terms]) OR ("Venous disease"[Title]) OR ("vein disease"[Title]) OR ("venous insufficiency"[Title]) OR ("vein insufficiency"[Title]) OR ("venous disorder"[Title]) OR ("vein disorder"[Title]) OR ("lower extremities"[Title]) OR ("Varicosity"[Title]) OR ("venous ulcer"[Title])))

AND

((("heart failure"[MeSH Terms]) OR ("heart failure"[Title]) OR ("myocardial infarction"[MeSH Terms]) OR ("myocardial infarction"[Title]) OR ("stroke"[MeSH Terms]) OR ("stroke"[Title]) OR ("ischemic disease"[Title]) OR ("coronary artery disease"[MeSH Terms]) OR ("coronary artery disease"[Title]) OR ("peripheral artery disease"[Title]) OR ("cardiac"[Title]) OR ("cardiovascular"[Title]) OR ("pulmonary embolism"[MeSH Terms]) OR ("pulmonary embolism"[Title]) OR ("venous thromboembolism"[Title]) OR ("mortality"[Title]) OR ("atrial fibrillation"[Title]) OR ("arterial disease"[Title])))

## Table S2.

Meta-analysis of the association between chronic venous disease and cardiovascular events (9 studies; 15 outcomes).^1-9^

| **Study** | | **Weights (%)** | **Number of patients (N)** | **HR (95% CI)** | **p-value** |
| --- | --- | --- | --- | --- | --- |
| Chang 2018^1^ | (VV) - DVT | 7.5% | 212,984 | 5.4 (5.2, 5.6) |  |
|  | (VV) - PE | 7.4% | 212,984 | 1.8 (1.6, 1.9) |  |
|  | (VV) - PAD | 7.5% | 212,984 | 1.8 (1.7, 1.8) |  |
| Choi 2024^2^ | (VV) - AF | 5.5% | 20,736 | 1.2 (1.1, 1.3) |  |
| Hsieh 2025^3^ | (VV) - STROKE | 5.5% | 23,238 | 1.2 (1.1, 1.2) |  |
| Hu 2022^4^ | (VV) - AF | 5.4% | 5579 | 1.2 (1.0, 1.5) |  |
| Kim 2025^5^ | (VV) - HF | 5.5% | 5004 | 1.2 (1.1, 1.3) |  |
| Mäkivaara 2007^6^ | (VV) - ARTDIS | 5.6% | 888 | 2.0* (1.5, 2.7) |  |
| Mäkivaara 2009^7^ | (VV) - HF | 4.0% | 989 | 2.1* (1.1, 4.0) |  |
| Rahman 2011^8^ | (VV) - CHD | 8.2% | 2713 | 0.9* (0.9, 0.9) |  |
|  | (VV) - HF | 8.2% | 1379 | 1.0* (1.0, 1.1) |  |
|  | (VV) - STROKE | 8.1% | 1116 | 0.9* (0.9, 1.0) |  |
|  | (VV) - PE | 8.0% | 524 | 1.1* (1.0, 1.2) |  |
|  | (VV) - DVT | 8.0% | 458 | 1.2* (1.1, 1.3) |  |
| Wu 2020^9^ | (VV) - MACE | 5.5% | 4644 | 2.0 (1.9, 2.2) |  |
| **Random-effects model** | | **100%** |  | **1.5 (1.1, 2.0)** | **0.006** |
| **Random-effects model adjusted^a^** | |  |  |  |  |
| Heterogeneity, *Q* = 7427.17, df = 14 (p<0.001 (S)); *I^2^* = 95.10%, tau^2^ = 0.20 | | | | | |
| Fixed effect model  Estimate = 1.7 (1.7, 1.8)  p-value <0.001 | | | | | |

* IRR approximated as HR.

^a^Results are not presented because no covariate was statistically significant or not enough data.

AF, atrial fibrillation; ARTDIS, arterial disease; CHD, coronary heart disease; CI, confidence interval; DVT, deep vein thrombosis; HF, heart failure; HR, hazard ratio; IRR, incidence rate ratio; MACE, major adverse cardiovascular events; PAD, peripheral artery disease; PE, pulmonary embolism; VV, varicose veins.

## Table S3.

Meta-analysis of the association between chronic venous disease and all-cause mortality (5 studies, 5 outcomes).^9-12^

| **Study** | | **Weights (%)** | **Number of patients (N)** | **Number of deaths (n)** | **HR (95% CI)** | **p-value** |
| --- | --- | --- | --- | --- | --- | --- |
| Kreft 2020^10^ | (VLU (C6)) | 25.7% | 4673 | NA | 1.1 (1.0, 1.1) |  |
| Prochaska 2021(a)^11^ | (CVI (C3–C6)) | 16.8% | 4603 | NA | 1.5 (1.2, 1.8) |  |
| Prochaska 2021(b)^11^ | (CVI (C3–C6 in MyoVasc study)) | 11.6% | 905 | NA | 1.5 (1.1, 2.1) |  |
| Salenius 2021^12^ | (VLU (C6)) | 25.1% | 1924 | 1526 | 1.5 (1.4, 1.6) |  |
| Wu 2020^9^ | (VV) | 20.9% | 4644 | 301.86 | 1.4 (1.2, 1.6) |  |
| **Random-effects model** | | **100%** |  |  | **1.3 (1.2, 1.6)** | **<0.001** |
| **Random-effects model adjusted^a^** | | **100%** |  |  |  |  |
| Heterogeneity, *Q =* 78.91, df = 4 (p<0.001 (S)); *I^2^* = 90.33%, tau^2^ = 0.02 | | | | | | |
| Fixed effect model   Estimate = 1.2 (1.2, 1.3)  p-value <0.001 | | | | | | |

^a^Results are not presented because no covariate was statistically significant or not enough data.

CI, confidence interval; CVI, chronic venous insufficiency; HR, hazard ratio; VLU, venous leg ulcer; VV, varicose veins.

## Table S4.

Meta-analysis of the association between chronic venous disease and coronary artery and heart disease (6 studies; 7 outcomes).^2,4,5,7-9^

| **Study** | | **Weights (%)** | **Number of patients (N)** | **HR (95% CI)** | **p-value** |
| --- | --- | --- | --- | --- | --- |
| Choi 2024^2^ | (VV) - AF | 6.7% | 20,736 | 1.2 (1.1, 1.3) |  |
| Hu 2022^4^ | (VV) - AF | 6.0% | 5579 | 1.2 (1.0, 1.5) |  |
| Kim 2025^5^ | (VV) - HF | 6.6% | 5004 | 1.2 (1.1, 1.3) |  |
| Mäkivaara 2009^7^ | (VV) - HF | 2.1% | 989 | 2.1* (1.1, 4.0) |  |
| Rahman 2011^8^ | (VV) - HF | 6.8% | 1379 | 1.0* (1.0, 1.1) |  |
| Wu 2020^9a^ | (VV) - ACS | 34.7% | 4644 | 1.8 (1.5, 2.1) |  |
|  | (VV) - HF | 37.1% | 4644 | 1.8 (1.6, 2.0) |  |
| **Random-effects model** | | **100%** |  | **1.3 (1.1, 1.6)** | **0.006** |
| **Random-effects model adjusted^b^** | |  |  |  |  |
| Heterogeneity, *Q* = 112.63, df = 6 (p<0.001 (S)); *I^2^* = 71.11%, tau^2^ = 0.05 | | | | | |
| Fixed effect model   Estimate = 1.2 (1.2, 1.2)  p-value <0.001 | | | | | |
| * IRR approximated as HR. | | | | | |

^a^HRs by VV grade were pooled using a fixed-effect meta-analysis on log-transformed HRs, with weights derived from CIs. The standard error was estimated using standard meta-analytic methods.

^b^Results are not presented because no covariate was statistically significant or not enough data

ACS, acute coronary syndrome; AF, atrial fibrillation; CI, confidence interval; HF, heart failure; HR, hazard ratio; IRR, incidence rate ratio; VV, varicose veins.

## Figure S1.

Quality assessment using the Newcastle-Ottawa Scale^13,14^ (13 studies).^1-12^

## Figure S2. Funnel plot assessing the publication bias of the meta-analysis of hazard ratios in cardiovascular events (9 studies; 15 outcomes).^1-9^

Each dot represents a cardiovascular event outcome from one of the nine included studies. As some studies contributed multiple outcomes, the observations are not fully independent, reflecting both within- and between-study variation. The funnel plot shows an overall symmetric distribution, while a slight asymmetry cannot be excluded, the overall pattern remains broadly consistent with a balanced distribution of effects.

## Figure S3.

Forest plot of the association between chronic venous disease and cardiovascular events, sensitivity analysis restricted to studies reporting hazard ratios (6 studies; 8 outcomes).^1-5,9^

AF, atrial fibrillation; CI, confidence interval; DVT, deep vein thrombosis; HF, heart failure; MACE, major adverse cardiovascular events; PAD, peripheral arterial disease; PE, pulmonary embolism; VV, varicose veins.

## Figure S4. Forest plot showing the hazard ratios of the association between chronic venous disease and cardiovascular events, sensitivity analysis removing outcome-specific estimates for deep vein thrombosis and pulmonary embolism (9 studies; 11 outcomes).^1-9^

AF, atrial fibrillation; ARTDIS, arterial disease; CHD, coronary heart disease; CI, confidence interval; HF, heart failure; MACE, major adverse cardiovascular events; PAD, peripheral artery disease; VV, varicose veins.

## Figure S5.

Funnel plot assessing the publication bias of the meta-analysis of hazard ratios in all-cause mortality (5 studies; 5 outcomes).^9-12^

Each dot represents an event outcome reported within one of the five included studies. The plot appears to be symmetrical.

## Figure S6.

Forest plot showing the hazard ratios^a^ for the association between chronic venous disease and coronary artery and heart disease (6 studies; 7 outcomes).^2,4,5,7-9^

^a^In two studies, values for IRRs were approximated as hazard ratios.^7,8^

ACS, acute coronary syndrome; AF, atrial fibrillation; CI, confidence interval; HF, heart failure; VV, varicose veins.

## Figure S7.

Funnel plot of the meta-analysis of hazard ratios in coronary artery and heart disease (6 studies; 7 outcomes).^2,4,5,7-9^

Each dot represents an event outcome reported within one of the six included studies. Because Wu 2020^9^ contributed to multiple outcomes, dots are not statistically independent. The funnel plot therefore reflects both within‑ and between‑study variation. No asymmetry was observed on visual inspection of the funnel plot.

# References

1.	Chang SL, Huang YL, Lee MC, Hu S, Hsiao YC, Chang SW, et al. Association of varicose veins with incident venous thromboembolism and peripheral artery disease. *JAMA* 2018;319:807-817.

2.	Choi S, Leem GH, Song TJ. Association of varicose veins with incidence risk of atrial fibrillation: a population-based cohort study. *Int J Surg* 2024;110:5704-5712.

3.	Hsieh FR, Lee WK, Chen SS, Sun CK, Yeh WB, Hung YM, et al. Varicose vein is associated with an increased risk of ischemic stroke: a population-based matched cohort study. *Postgrad Med J* 2025;101:1173-1180.

4.	Hu WS, Lin CL. Association between varicose vein and atrial fibrillation-a population-based study in Taiwan. *Phlebology* 2022;37:535-539.

5.	Kim D, Park MS, Park JY, Song TJ. Association of varicose veins with the risk of heart failure: A nationwide cohort study. *PLoS One* 2025;20:e0316942.

6.	Mäkivaara LA, Ahti TM, Luukkaala T, Hakama M, Laurikka JO. Persons with varicose veins have a high subsequent incidence of arterial disease: a population-based study in Tampere, Finland. *Angiology* 2007;58:704-709.

7.	Mäkivaara LA, Ahti TM, Luukkaala T, Hakama M, Laurikka JO. The risk of congestive heart failure is increased in persons with varicose veins. *Int Angiol* 2009;28:452-457.

8.	Rahman F, Wotton CJ, Goldacre MJ. Varicose veins, haemorrhoids and the risk of circulatory diseases: record-linkage study. *Br J Cardiol* 2011;18:124–129.

9.	Wu NC, Chen ZC, Feng IJ, Ho CH, Chiang CY, Wang JJ, et al. Severe varicose veins and the risk of mortality: a nationwide population-based cohort study. *BMJ Open* 2020;10:e034245.

10.	Kreft D, Keiler J, Grambow E, Kischkel S, Wree A, Doblhammer G. Prevalence and mortality of venous leg diseases of the deep veins: an observational cohort study based on German health claims data. *Angiology* 2020;71:452-464.

11.	Prochaska JH, Arnold N, Falcke A, Kopp S, Schulz A, Buch G, et al. Chronic venous insufficiency, cardiovascular disease, and mortality: a population study. *Eur Heart J* 2021;42:4157-4165. (written Prochaska (a) and (b))

12.	Salenius JE, Suntila M, Ahti T, Huhtala H, Vaalasti A, Salmi TT, et al. Long-term mortality among patients with chronic ulcers. *Acta Derm Venereol* 2021;101:adv00455.

13.	Wells G, Shea B, O'Connell D, Peterson J, Welch V, Losos M, et al. *Newcastle-Ottawa quality assessment scale: case-control studies*. <https://ohri.ca/en/who-we-are/core-facilities-and-platforms/ottawa-methods-centre/newcastle-ottawa-scale> (accessed 16 March 2026)

14.	Stang A. Critical evaluation of the Newcastle-Ottawa scale for the assessment of the quality of nonrandomized studies in meta-analyses. *Eur J Epidemiol* 2010;25:603-605.
